# NASH, Fibrosis and Hepatocellular Carcinoma: Lipid Synthesis and Glutamine/Acetate Signaling

**DOI:** 10.3390/ijms21186799

**Published:** 2020-09-16

**Authors:** Yoshiaki Sunami

**Affiliations:** Department of Visceral, Vascular and Endocrine Surgery, Martin-Luther-University Halle-Wittenberg, University Medical Center Halle, 06120 Halle, Germany; yoshiaki.sunami@uk-halle.de; Tel.: +49-345-557-2794

**Keywords:** NASH, NAFLD, liver fibrosis, hepatocellular carcinoma, lipid metabolism, glutamine metabolism, acetate metabolism

## Abstract

Primary liver cancer is predicted to be the sixth most common cancer and the fourth leading cause of cancer mortality worldwide. Recent studies identified nonalcoholic fatty liver disease (NAFLD) as the underlying cause in 13–38.2% of patients with hepatocellular carcinoma unrelated to viral hepatitis and alcohol abuse. NAFLD progresses to nonalcoholic steatohepatitis (NASH), which increases the risk for the development of liver fibrosis, cirrhosis, and hepatocellular carcinoma. NAFLD is characterized by dysregulation of lipid metabolism. In addition, lipid metabolism is effected not only in NAFLD, but also in a broad range of chronic liver diseases and tumor development. Cancer cells manipulate a variety of metabolic pathways, including lipid metabolism, in order to build up their own cellular components. Identifying tumor dependencies on lipid metabolism would provide options for novel targeting strategies. This review article summarizes the research evidence on metabolic reprogramming and focuses on lipid metabolism in NAFLD, NASH, fibrosis, and cancer. As alternative routes of acetyl-CoA production for fatty acid synthesis, topics on glutamine and acetate metabolism are included. Further, studies on small compound inhibitors targeting lipid metabolism are discussed. Understanding reprogramming strategies in liver diseases, as well as the visualization of the metabolism reprogramming networks, could uncover novel therapeutic options.

## 1. Introduction

Primary liver cancer is predicted to be the sixth most common cancer and the fourth leading cause of cancer mortality worldwide in 2018 [1]. Hepatocellular carcinoma (HCC) is the most prevalent primary liver cancer and accounts for 90% of cases, followed by intrahepatic cholangiocarcinoma [2]. HCC usually develops within a background of advanced chronic liver diseases, such as chronic infection with hepatitis B virus (HBV), hepatitis C virus (HCV), and alcohol abuse. Recent studies identified nonalcoholic fatty liver disease (NAFLD) as the underlying cause in 13–38.2% of patients with HCC unrelated to virus and alcohol [3]. NAFLD is tightly associated with the metabolic syndrome, and metabolic reprogramming has also been firmly recognized as a hallmark of cancer [4]. To that end, metabolic reprogramming plays key roles during disease progression and in the cancer stage. Cancer cells reprogram a variety of metabolic pathways in order to build up their own cellular components, such as nucleic acids, proteins, and lipids. Activation of lipid synthesis is highly important for rapidly growing cancer cells, because lipids such as phospholipid bilayers are fundamental membrane components enabling cellular proliferation [5]. In a wide variety of tumors, de novo synthesis of fatty acids (FAs) is activated irrespective of the levels of circulating lipids. It has been shown that several signaling pathways in cancer cells can activate de novo FA synthesis, covering more than 93% of triacylglycerol FAs [6]. The reprogramming of cellular metabolism during tumorigenesis can be a consequence of oncogenic mutations. The current review article provides a background of liver cancer metabolism, focusing on lipid metabolism and targeting strategies for modulating factors and enzymes associated with lipid metabolic pathways.

## 2. Fatty acid Synthesis and Targeting Strategies for Liver Diseases

**Figure 1:**
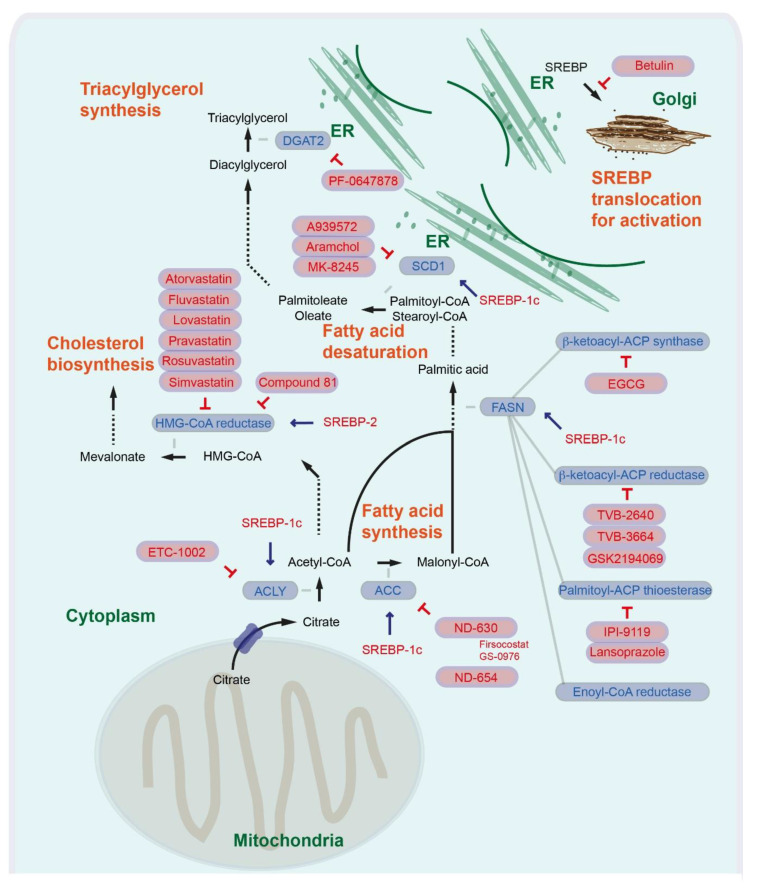
Regulation of fatty acid synthesis, fatty acid desaturation, triacylglycerol synthesis, cholesterol synthesis, and SREBP translocation. The inhibition symbols are colored in red.

In the first step of FA synthesis, cytoplasmic acetyl-CoA is generated from citrate by ATP-citrate lyase (ACLY), then acetyl-CoA carboxylase (ACC) catalyzes conversion into malonyl-CoA. Malonyl-CoA and acetyl-CoA are coupled to the acyl carrier protein (ACP) domain of the multienzyme protein fatty acid synthase (FASN). In an NADPH-dependent manner, repeated condensations of acetyl groups by the FASN lead to generation of palmitic acid, a basic 16-carbon saturated FA (Figure 1) [7]. In human cancer cells, expression of ACLY and ACC is also markedly increased [6]. When the cellular energy state is low, AMP-activated protein kinase (AMPK) phosphorylates and directly inhibits ACC1 and ACC2 (S80 for ACC1 and S222 for ACC2 in humans, S79 for ACC1 and S212 for ACC2 in mice [8]). In mice with targeted knock-in mutations in which the AMPK phosphorylation sites on ACC1/2 are converted to alanine, there is a loss of AMPK-mediated ACC inhibition, elevated hepatic lipogenesis, insulin resistance, and early signs of NAFLD and fibrosis [9]. Aberrant activation of FASN and de novo synthesis is a major metabolic event in HCC development. In human HCC, expression of major enzymes associated with de novo lipogenesis, including FASN, is increased in tumor lesions compared with liver non-neoplastic counterparts. Overexpression of Akt increases the expression of ACLY, ACC, and FASN in human cancer cells. Consistently, knockdown of Akt in human cancer cells leads to downregulation of ACLY, ACC, and FASN [10]. Overexpression of activated Akt and c-Met in the mouse liver by hydrodynamic transfection induces liver tumor development. Depletion of FASN in mice abolishes Akt/c-Met-induced hepatocarcinogenesis (Akt/c-Met and Cre plasmids were co-injected into *Fasn^lox/lox^* mice) [11]. Furthermore, conditional deletion of FASN (*Alb-Cre; Fasn^lox/lox^*) delays the hepatocarcinogenesis induced by loss of tumor suppressor *Pten* and overexpression of c-Met and prolongs survival in mice [12]. Targeting de novo FA synthesis by inhibiting ACLY, ACC, or FASN could be a therapeutic option for HCC.

Bempedoic acid (ETC-1002, 8-hydroxy-2,2,14,14-tetramethylpentadecanedioic acid) is an ACLY competitive inhibitor that also activates AMP-activated protein kinase (AMPK), which has been used in phase 3 clinical trials as a cholesterol-reducing agent (Figure 1) [13]. Several studies have shown that bempedoic acid has pharmacological effects, specifically in the liver [14]. Bempedoic acid forms bempedoic acid-CoA in the liver. For the conversion, the very long chain acyl-CoA synthetase 1 (ACSVL1) is required. ACSVL1 is expressed specifically in the liver of rodents and pigs, but not in the adipose tissue, intestinal muscle, or skeletal muscle [14,15]. In preclinical mouse models, administration of bempedoic acid (ETC-1002) (intraperitoneal injection) attenuates hepatotoxin diethylnitrosoamine (DEN) and high fat diet induced hepatocellular carcinogenesis [16]. For targeting of ACC, a series of potent allosteric protein–protein interaction inhibitors has been identified. These inhibitors interact within the ACC subunit phosphopeptide acceptor and dimerization site, leading to enzymatic activity inhibition [17]. ND-630 (1,4-dihydro-1-[(2*R*)-2-(2-methoxyphenyl)-2-[(tetrahydro-2*H*-pyran-4-yl)oxy]ethyl]-α,α,5-trimethyl-6-(2-oxazolyl)-2,4-dioxo-thieno[2,3-*d*]pyrimidine-3(2*H*)-acetic acid) given by oral gavage reduces high sucrose diet induced hepatic steatosis. Administration of ND-630 further reduces high fat diet induced hyperleptinemia, hyperinsulinemia, hepatic steatosis, hepatic cholesterol, and improves insulin sensitivity in rats [17]. ND-630 is also known as GS-0976 or firsocostat (Figure 1) and GS-0976 (Firsocostat) reduces hepatic de novo lipogenesis, hepatic steatosis, and fibrosis markers in patients with NASH (Table 1) (NCT02856555) [18,19]. A phase 2 study was completed to assess the safety and tolerability of selonsertib, firsocostat (GS-0976), and cilofexor administered alone or in combination in patients with bridging fibrosis or compensated cirrhosis due to NASH (Table 1) (NCT03449446). A phase 2 study to evaluate the safety and tolerability of selonsertib, firsocostat (GS-0976), cilofexor, fenofibrate, and vascepa in patients with NAFLD or NASH is currently ongoing (Table 1) (NCT02781584). ND-654 (2-(1-((R)-2-(((1s,4S)-4-hydroxycyclohexyl)oxy)-2-(2-methoxyphenyl)ethyl)-5-methyl-6-(oxazol-2-yl)-2,4-dioxo-1,4-dihydrothieno[2,3-d]pyrimidin-3(2H)-yl)-2-methylpropanoic acid) is another derivative that has been modified for enhanced hepatic uptake. Oral administration of ND-654 attenuates DEN-induced hepatocellular carcinogenesis in rats. Furthermore, ND-654 improves survival and the efficacy of Sorafenib in DEN-induced cirrhosis and HCC in rats (Figure 1) [20].

FASN is a multienzyme protein complex with two identical polypeptides; therefore, targeting FASN can be performed by several different approaches. The enzyme complex includes several catalytic domains with acyl carrier protein (ACP), malonyl/acetyltransferase (MAT), β-ketoacyl-ACP synthase, β-ketoacyl-ACP reductase, 3-hydroxyacyl-ACP dehydrase, enoyl-CoA reductase, and palmitoyl-ACP thioesterases. Several inhibitors have been suggested to block β-ketoacyl-ACP synthase of FASN, such as the small antibiotic molecule cerulenin ((2*S*,3*R*)-2,3-epox-4-oxo-7,10-dodecadienoylamide), the cerulenin-derived semisynthetic molecule with improved stability named C75 (4-methylene-2-octyl-5-oxotetrahydrofuran-3-carboxylic acid), and epigallocatechin gallate (EGCG). Inhibition of FASN with EGCG has been considered for several cancer types, including prostate, lung, breast, and colorectal cancer, for which several phase 2 and phase 3 clinical trials are ongoing [5]. For liver cancer, a phase 1 study with catechin is ongoing to see how well it works in preventing liver cancer in patients with cirrhosis (Table 1) (NCT03278925). The β-lactone orlistat blocks palmitoyl-ACP thioesterase [6]. However, there are some limitations with cerulenin, C75, and orlistat due to off-target toxicity and tissue distribution [21]. Several compounds such as TVB-2640 (also known as ASC40) (4-(1-(4-Cyclobutyl-2-methyl-5-(5-methyl-4H-1,2,4-triazol-3-yl)benzoyl)piperidin-4-yl)benzonitrile), IPI-9119 (4-(4-(2,6-difluorophenyl)-N-isopropyl-5-oxo-4,5-dihydro-1H-tetrazole-1-carboxamido)-3-phenoxybenzoic acid), and GSK2194069 (4-[4-(5-benzofuranyl)phenyl]-5-[[(3S)-1-(cyclopropylcarbonyl)-3-pyrrolidinyl]methyl]-2,4-dihydro-3H-1,2,4-triazol-3-one) have been proposed. IPI-9119 is an irreversible palmitoyl-ACP thioesterase inhibitor, while IPI-9119 antagonizes prostate cancer growth xenografts and human prostate cancer derived organoids [22]. GSK2194069 is a potent and specific inhibitor of the β-ketoacyl-ACP reductase activity [23]. TVB-2640 also inhibits the β-ketoacyl-ACP reductase activity (NCT02980029). An analog of the drug, TVB-3664 (4-(1-(5-(4-(methoxymethyl)-2-(trifluoromethyl)-1H-imidazol-5-yl)-2,4-dimethylbenzoyl)azetidin-3-yl)benzonitrile), has also been considered for cancer therapy. Oral administration with TVB-3664 inhibits tumor growth in colorectal cancer patient derived xenografts in mice and attenuates Akt and Erk signaling activity [24]. TVB-2640 reduces hepatic de novo lipogenesis in patients with NAFLD and NASH [25]. Currently, a phase 2 study is recruiting subjects with NASH to evaluate the safety and efficacy of TVB-2640 (Table 1) (NCT03938246). Further, in silico screening of FDA-approved drugs has identified alternative inhibitors of the thioesterase domain. The proton pump inhibitors lansoprazole, rabeprazole, omeprazole, and pantoprazole function as inhibitors of thioesterase activity, which can induce pancreatic cancer cell death [26]. Lansoprazole has been shown to prevent progression of liver fibrosis in a choline-deficient, amino-acid-defined (CDAA), diet-induced NASH model in rats [27]. Proton pump inhibitors have been suggested to increase the risk for hepatic encephalopathy in patients with cirrhosis [28,29]. The safety and efficacy of proton pump inhibitors as inhibitors of thioesterase activity in patients with liver diseases still need to be evaluated (Figure 1).

## 3. Fatty Acid Desaturases and Deacylglycerol Acyltransferase in Liver Diseases

The main product of fatty acid synthesis in the cytoplasm is 16-carbon saturated palmitic acid. On the cytosolic side of the endoplasmic reticulum (ER), longer FAs are formed. In mammalian cells, several types of fatty acid desaturases introduce carbon double bonds at Δ^5^ (D5D), Δ^6^ (fatty acid desaturase 2, FADS2, D6D), or Δ^9^ (Δ^9^-stearoyl-CoA desaturase) (SCD). SCD is the rate-limiting enzyme catalyzing the synthesis of monounsaturated 16- or 18-carbon-like palmitoleate and oleate from palmitoyl-CoA and stearoyl-CoA [5,30]. In the nonalcoholic human fatty liver, SCD1 activity and diacylglycerol are increased [31]. Humans express both *SCD1* and *SCD5*, of which *SCD1* is the main isoform. Deacylglycerol acyltransferase 2 (DGAT2) catalyzes the de novo synthesis of triglycerols from newly synthesized FAs (CoA + triacylglycerol from acyl-CoA + 1,2-diacylglycerol), while hepatic overexpression of DGAT2 in mice leads to hepatic steatosis [32,33,34]. Hepatic deletion of *Dgat2* in mice reduces fructose-palmitate-cholesterol (FPC)-diet-induced steatosis without increasing inflammation or fibrosis [35]. Therefore, the development of DGAT2 inhibitors could be a therapeutic strategy. Treatment with a selective DGAT2 inhibitor PF-06427878 (Figure 1) reduces hepatic and circulating plasma triglyceride concentrations in rats maintained on a Western-type diet (high fat, high cholesterol diet) and attenuates liver fibrogenesis in STAM mouse models (streptozotocin and high fat diet) of NASH-HCC. Several phase 1 studies have been completed to evaluate the safety and effect of PF-06427878 in healthy adults (Table 1) (NCT02391623; NCT02855177) [36].

SCD1 is overexpressed in diverse cancer types [30], while SCD also promotes liver fibrosis and tumor development in mice [37]. Expression of SCD1 is associated with shorter survival times for breast (relapse-free and overall), liver (disease-free), lung (3-year), pancreatic, and colorectal (overall) cancers [38,39,40,41,42]. Furthermore, SCD1 regulates endoplasmic reticulum (ER)-stress-mediated sorafenib resistance in liver cancer patients [41]. Expression of CD24, a cancer stem cell (CSC)-associated cell surface marker, correlates with sorafenib resistance and shorter overall survival [43]. SCD1 is involved in maintaining cancer cell stemness, while knockdown of SCD1 reduces the expression of *SOX2* and *NANOG*, which are other stemness markers [44]. Cancer stemness may be responsible not only for tumor initiation, but also for metastasis [45]. Targeting SCD1 could, therefore, be a promising therapeutic option; however, the role of SCD1 in animal models remains controversial and requires further investigation. SCD1 deficiency protects mice from high carbohydrate but not high fat diet induced adiposity [46]. SCD1 expression is, however, dispensable for hepatocellular carcinogenesis induced by hydrodynamic gene delivery of oncogenic Akt/Ras [47]. Conditional deletion of *Scd1* in the intestinal epithelium (*Vil1-Cre; Scd1^lox/lox^*) promotes inflammation and tumorigenesis driven by mutant allele multiple intestinal neoplasia (Min) of the adenomatous polyposis coli (Apc) locus (*Apc^Min/+^* mice) [48]. The mouse has 4 *Scd* genes (*Scd1*-*4*), of which *Scd1* is predominantly expressed in the adult liver and *Scd2* in the embryonic liver [49]. Mice with conditional disruption of *Scd2* in activated hepatic stellate cells (HSCs) (*Cola1a-Cre; Scd2^lox/lox^*) show reduced incidence rates for DEN-induced and Western alcohol diet promoted liver tumor [37]. Some cancer cells, including liver cancer cells, desaturate palmitoyl-CoA to generate sapienate via the Δ^6^ desaturase FADS2 and support membrane biosynthesis during proliferation. This metabolic plasticity involves metabolic rewiring and increases cancer plasticity [50].

However, several studies suggest that targeting SCD1 could still be a promising option. An SCD1 inhibitor *N*-(2-hydroxy-2-phenylethyl)-6-[4-(2-methyl benzoyl) piperidin-1-yl]pyridazine-3-carboxamide attenuates hepatic lipid accumulation and fibrosis in methionine- and choline-deficient (MCD) diet models [51]. Inhibition with another SCD1 inhibitor A939572 (piperidine-aryl urea-based molecules) sensitizes HCC cells to the effects of sorafenib [41]. Treatment with aramchol (arachidyl amido cholanoic acid) reduces MCD diet-induced steatohepatitis and liver fibrosis [52]. A phase 2 and 3 study with aramchol in patients with NASH has been completed (Table 1) (NCT02279524). Currently a phase 3 and 4 study in subjects with NASH is ongoing (Table 1) (NCT04104321). As a liver-specific SCD1 inhibitor, MK-8245 (5-[3-[4-(2-bromo-5-fluorophenoxy)-1-piperidinyl]-5-isoxazolyl]-2H-tetrazole-2-acetic acid) was designed by utilizing liver-specific, organic-anion-transporting polypeptides (Figure 1) [53]. A phase 1 study assessing the safety, tolerability, pharmacokinetics, and glucose-lowering activity of MK-8245 in participants with type 2 diabetes has been completed (Table 1) (NCT00972322). The effects of these inhibitors on liver cancer are currently not known.

## 4. Sterol Regulatory Element-Binding Protein in Liver Diseases—Master Regulators of Fatty Acids and Cholesterol Synthesis

The transcription factor sterol regulatory element-binding protein 1c (SREBP-1c) regulates expression of genes involved in FA synthesis and modifications such as *ACLY, FASN and SCD1* as well as *ACACA/B*, which code ACC1 and ACC2, respectively. Several signaling pathways and factors such as PI3K/Akt and mammalian target of rapamycin (mTOR) complex 1 (mTORC1) regulate SREBP-1c activity [54]. There are several SREBP isoforms, including SREBP-1a, SREBP-1c, and SREBP-2. Both SREBP-1a and SREBP-1c are derived from a single gene, however through different transcription start sites. Whereas SREBP-1c preferentially regulates genes involved in FA synthesis, SREBP-1a is suggested to be a potent activator of all SREBP-responsive genes, while SREBP-2 is more restricted to regulating cholesterol biosynthesis [55]. SREBPs interact with the SREBP cleavage-activating protein (SCAP), and the SREBP/SCAP complex further consists of the ER membrane proteins insulin-induced gene 1 (INSIG1) and INSIG2. Under physiological conditions, reduction of cellular lipid levels triggers conformational changes of SCAP, abrogating its interactions with INSIGs. Subsequently, dissociation of the SREBP/SCAP complex from INSIGs leads to translocation from the ER to the Golgi, where SREBP is cleaved and activated [56].

Oncogenic PI3K (H1047R mutation) and Kras (G12V mutation) are able to induce de novo lipid synthesis and expression of the *SREBF1* gene (coding SREBP-1) [57]. SREBP-1 is upregulated in HCC patient tissues. The positive expression of SREBP-1c correlates with a shorter 3-year overall and disease-free survival of HCC patients [58]. High SREBP-1 expression also correlates with a shorter overall survival of HCC patients who received sorafenib treatment [59]. Targeting SREBP-1c may be a therapeutic option for HCC patients. Betulin directly binds to the SCAP, therefore inhibiting SREBP-1 activation, and improves hyperlipidemia and insulin resistance (Figure 1) [60]. Betulin enhances the antitumor effects of sorafenib on HCC cells and xenograft liver tumor growth [59]. Consistently, hepatocyte-specific conditional knockout of Scap decreases DEN-induced hepatocarcinogenensis [61]. Taken together, these results show that the targeting of SREBP-1 is a promising option for liver cancer therapy. The limitation in clinical application for cancer therapy with betulin is its poor solubility in aqueous media; therefore, generating betulin derivatives with higher solubility may be important [62].

Cholesterol is an essential structural constituent of cell membranes, together with various phospholipids, sphingomyelin, and glycolipids [63]. Cholesterol is synthesized de novo from cytoplasmic acetyl-CoA through the mevalonate pathway. The rate-limiting step is the conversion of 3-hydroxy-3-methylglutaryl-CoA (HMG-CoA) to mevalonate by HMG-CoA reductase (HMGCR) [64]. Beyond de novo cholesterol synthesis, cells can also increase their cholesterol contents through receptor-mediated endocytosis of low-density lipoproteins (LDLs) [65]. HMG-CoA reductase and the LDL receptor (LDLR) are both transcriptional targets of SREBP-2 [55]. Tumor suppressor P53-binding protein 2 (TP53BP2, also known as apoptosis-stimulating p53 protein 2, ASPP2), a p53 activator, negatively regulates the mevalonate pathway and inhibits the growth of HCC cells, indicating that activation of the mevalonate pathway can play an important role in liver cancer development. In line with this, patients with high TP53BP2 expression and low HMG-CoA reductase expression in the liver show longer overall survival and recurrence-free survival [66]. Inhibiting the cholesterol synthesis pathway by blockage of the rate-limiting enzyme HMG-CoA reductase has also been considered for cancer therapy. Statins act by competitively binding to the catalytic domain of HMG-CoA reductase and blocking the conversion of HMG-CoA to mevalonate [67]. Atorvastatin and fluvastatin dose-dependently reduces cirrhosis and HCC in patients with hepatitis C virus [68]. Several statin derivatives (Figure 1) such as atorvastatin, lovastatin, pravastatin, rosuvastatin, and simvastatin have entered clinical trials. A phase 4 study with atorvastatin is ongoing for HCC patients (Table 1) (NCT03024684). A combination trial with atorvastatin and sorafenib for HCC patients is currently ongoing (phase 2) (Table 1) (NCT03275376). A phase 3 study with sorafenib with and without pravastatin has been completed and a phase 2 study to evaluate whether pravastatin intervention can delay or protect against HCC recurrence is currently ongoing (Table 1) (NCT01075555; NCT03219372). Simvastatin has also been considered for preventing liver cancer in patients with liver cirrhosis, and a phase 2 study is currently ongoing (Table 1) (NCT02968810). Taken together, the result show that inhibition of HMG-CoA reductase by statin derivatives is highly attractive as a single or combinational therapeutic option in liver cancer therapy.

A drawback of statins is that they induce compensatory increases of HMG-CoA reductase. Mice gavaged with lovastatin led to high hepatic HMG-CoA reductase expression [69]. Mice treated with rosvastatin and storvastatin also showed high hepatic HMG-CoA reductase levels. Treatment with statins induces gene expression of the cholesterol synthesis enzymes *Mvk* (mevalonate kinase, which converts mevalonate into mevalonate-5-phosphate), *Pmvk* (phosphomevalonate kinase, which converts mevalonate-5-phosphatate into mevalonate-5-pyrophosphate), *Fdft1* (Farnesyl-diphosphate farnesyltransferase, also known as squalene synthase, which converts farnesyl pyrophosphate into squalene), and *Sqle* (squalene epoxidase also called squalene monooxygenase, which oxidizes squalene to 2,3-oxidosqualene/squalene epoxide) [70]. These compensatory feedback regulations hamper the effectiveness of the drugs. To solve the problem, a potent HMG-CoA reductase degrader has been generated (named compound 81), which eliminates statin-induced reductase accumulation and lowers cholesterol (Figure 1) [69]. Inducing HMG-CoA reductase degradation using compound 81 or other chemicals could be a strategy to improve statin therapy for the treatment of liver cancer. Compensatory activation of cholesterol biosynthesis is also triggered by FASN deletion. Loss of Fasn in Pten/c-Met-induced murine HCC cells leads to elevated expression of cholesterol biosynthesis genes, including *Hmgcr*, resulting in high cholesterol ester levels. Co-expression of dominant negative Srebp2 completely blocks Pten/c-Met-induced HCC formation in Fasn^LKO^ mice. This suggests that concomitant inhibition of FASN-mediated de novo FA synthesis and the mevalonate-pathway-mediated cholesterol biosynthesis could be a therapeutic option for liver cancer [12].

## 5. Glutamine Metabolism as a Metabolic Detour for Liver Diseases

**Figure 2:**
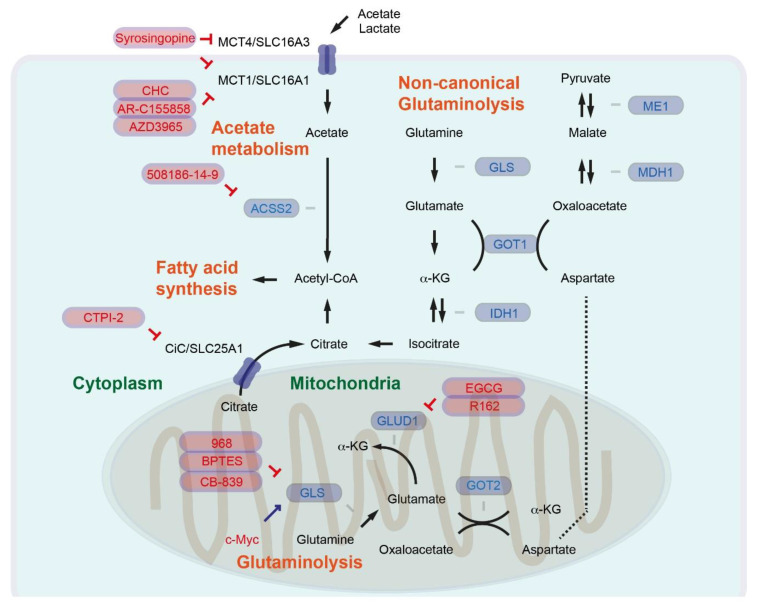
Regulation of glutaminolysis and acetate metabolism. The inhibition symbols are colored in red.

Cancer cells reprogram the activity of several metabolic pathways, including glutamine metabolism, to enable continuous production of FAs necessary for cell growth. Glutamine is the most abundant and nonessential amino acid that can be synthesized from glucose. In the canonical pathway of mitochondrial glutamine catabolism (glutaminolysis) (Figure 2), glutaminase (GLS) catalyzes glutamine to glutamate. Glutamate dehydrogenase (GLUD1) catalyzes a further conversion from glutamate to α-ketoglutarate (α-KG), then α-KG can be incorporated into the tricarboxylic acid cycle (TCA cycle) [5]. Glutamine has been shown to be an essential nutrient for the proliferation of human cancer cells [71], and glutamine synthetase (conversion of glutamate to glutamine) is overexpressed in human primary liver cancer [72]. GLS1, which is expressed in the mitochondrial matrix, is also upregulated in HCC. *GLS1* mRNA can give rise to two isoforms, including the longer form KGA and the shorter form GAC. High expression of the GAC isoform combined with low KGA expression causes shorter overall survival [73]. The Hedgehog–YAP signaling pathway regulates glutaminolysis, supporting activation of hepatic stellate cells [74]. Oncogenic c-Myc enhances the expression of mitochondrial GLS for canonical glutaminolysis [75].

The predicted cellular localization of glutaminase isozymes has been discussed elsewhere [76]. In the case of pancreatic cancer, it has been shown that a cytoplasmic and so-called noncanonical glutaminolysis pathway producing pyruvate via aspartate aminotransaminase (AST1, or glutamic oxaloacetic transaminase GOT1, which catalyzes aspartate, α-ketoglutarate or oxaloacetate, and glutamate), malate dehydrogenase (MDH1, which catalyzes malate or oxaloacetate), and malate enzyme (ME1, which catalyzes malate or pyruvate) (Figure 2). By inhibiting mitochondrial GLUD1 and activating cytoplasmic GOT1, oncogenic KRAS switches from canonical to noncanonical glutaminolysis [77]. By reprogramming the glutamine metabolism from the mitochondrial to the cytoplasmic system, pancreatic cancer can keep the synthesis of FAs intact, because cytoplasmic isocitrate dehydrogenase (IDH1) can catalyze α-KG/isocitrate under hypoxic conditions, or even with defective mitochondria [78,79,80]. It has not been fully understood whether other oncogenic signaling pathways can also support the shift from canonical to noncanonical glutaminolysis, or whether liver cancer also reprograms glutamine metabolism in general. One study, however, suggested that peroxisome-proliferator-activated receptor-γ (PPARγ) can reprogram glutamine metabolism in sorafenib-resistant HCC [81].

For the targeting pf GLS, several inhibitors have been developed, such as compound 968 (5-(3-Bromo-4-(dimethylamino)phenyl)-2,2-dimethyl-2,3,5,6-tetrahydrobenzo[a]phenanthridin-4(1H)-one), bis-2-(5-phenylacetamido-1,2,4-thiadiazol-2-yl)ethyl sulfide (BPTES), and molecule CB-839 (2-(pyridin-2-yl)-N-(5-(4-(6-(2-(3-(trifluoromethoxy)phenyl)acetamido)pyridazin-3-yl)butyl)-1,3,4-thiadiazol-2-yl)acetamide) (Figure 2) [82]. Treatment of myofibroblastic hepatic stellate cells (HSCs) with BPTES or CB-839 suppresses HSC growth [74]. In an inducible Myc-mediated model of HCC, mice expressing the tetracyclin-responsive transactivator (tTA) under the control of the liver-enriched transcriptional activator protein (LAP) promotor are crossed with mice bearing *Myc* under the control of a tTA-regulated promotor (named here LAP-tTA/Myc mice). Mice derived from crosses of LAP-tTA/Myc mice with *Gls* (*Gls^+/-^*) heterozygotes show delayed Myc-induced liver tumorigenesis, and treatment with BPTES results in prolonged survival of LAP-tTA/Myc animals [83]. CB-839 has already been tested in clinical studies for several cancer types. However, oral gavage of CB-839 exhibits no antitumor activity in mice with pancreas-specific (*Pdx1-Cre*) expression of oncogenic KRAS^G12D^ combined with Trp53 heterozygosity (*Trp53^lox/+^*). In addition, mice treated with CB-839 show marginally shorter survival than the group without CB-839 treatment [84]. Further investigations are needed to evaluate whether GLS inhibition is a potent therapeutic option for pancreatic and liver cancer patients.

EGCG has been suggested to block β-ketoacyl-ACP synthase of FASN (see above), but is also considered to inhibit GLUD1. A purpurin analog R162 (Figure 2) has been identified as a potent and specific GLUD1 inhibitor from a group of purpurin derivatives. R162 attenuated cell viability in a group of human lung cancer, breast cancer, and leukemia cell lines [85]. GLUD1 inhibition might be ineffective in pancreatic cancer, since oncogenic KRAS^G12D^ has been suggested to inhibit GLUD1 and preferentially activate the noncanonical glutaminolysis pathway. The effectiveness of GLUD1 inhibition in liver cancer may also depend on the reprograming of glutamine metabolism.

Pyruvate generated via noncanonical glutaminolysis or glycolysis can be transported to mitochondria and converted into acetyl-CoA by the pyruvate dehydrogenase complex. Citrate synthase catalyzes the conversion of mitochondrial acetyl-CoA into citrate. Citrate is then transported from the mitochondria to the cytosol by the citrate carrier (CiC, SLC25A1), and as mentioned above ACLY further catalyzes citrateto acetyl-CoA [5]. Citrate synthase is upregulated in HCC tumors [86]. The livers of patients with NASH show higher expression levels of CiC/SLC25A1 relative to normal livers. Furthermore, in mice fed with HFD, CiC/SLC25A1 expression is increased in the liver relative to control mice. The liver-specific deletion of *Slc25a1* (*Alb-Cre; Slc25a1^lox/lox^*) leads to attenuation of HFD-induced steatosis. Inhibition of CiC/SLC25A1 with a specific inhibitor compound CTPI-2 (2-((4-chloro-3-nitrophenyl)sulfonamido)benzoic acid) (Figure 2) reverses steatosis, glucose intolerance, and inflammation in HFD-fed mice [87].

## 6. Acetate Metabolism Is an Alternative Route to Provide Acetyl-CoA

Acetyl-CoA represents a central metabolite involved in regulating gene expression as a key determinant of protein and histone acetylation [88,89]. Cells with ACLY deficiency remain viable and proliferate, suggesting that citrate is not the unique metabolite of acetyl-CoA. Additional or alternative sources of acetyl-CoA could still be necessary for sufficient support of lipid synthesis and cancer cell growth. A total of 26 acyl-CoA synthetases (ACS) have been identified in the human genome. Three enzymes belonging to the short-chain ACS (ACSS) family (acetyl-CoA synthetases) are capable of catalyzing the synthesis of acetyl-CoA from acetate in an ATP-dependent manner [90]. ACSS1 and ACSS3 are mitochondrial enzymes, whereas ACSS2 localizes to both the nucleus and cytoplasm. Loss of ACSS2 suppresses tumor development in certain mouse liver cancer models, including c-Myc expression combined with PTEN knockout in the liver (*Alb-c-Myc; Alb-Cre; Pten^lox/lox^*). Acetate uptake correlates with ACSS2 expression in the HCC of liver cancer models [91]. Loss of ACSS2 protects against HFD-induced lipid deposition and obesity in mice [92]. Fructose intake triggers de novo lipogenesis in the liver and has been shown to be ACLY-independent. Acetyl-CoA for de novo lipogenesis is preferentially produced from acetate in hepatocytes, while silencing of hepatic *Acss2* using an adeno-associated viral (AAV) hairpin targeting *Acss2* suppresses FA synthesis from fructose [93].

Inhibitors specifically targeting ACSS2 remain largely unexplored. So far, the compound 1-(2,3-di(thiophen-2-yl)quinoxalin-6-yl)-3-(2-methoxyethyl)urea (known as 508186-14-9) (Figure 2) has been proposed as a ACSS2-specific inhibitor [91]. Targeting *ACSS2* and acetate metabolism would be a highly interesting concept for treating liver cancer.

In addition to fructose, acetate-containing foods such as processed meats, ethanol involved in oxidative catabolism, and indigestible carbohydrates have been considered as the major dietary sources of acetates [94]. The transporters involved in acetate uptake in cancer cells remain largely unexplored. Monocarboxylate transporters 1 and 4 (MCT1, MCT4), also known as SLC16A1 and SLC16A3, respectively, comprise the solute carrier (SLC) group of membrane transport proteins. MCT1 and MCT4 are generally involved in the transport of lactate, pyruvate, and ketone bodies, but are also involved in acetate transport [95]. It has been shown that MCT1 and MCT4 are expressed in many types of cancer [94]. HCC cell lines with high acetate uptake show higher MCT1 expression. Further MCT1 expression is elevated in human HCCs with high acetate uptake [96]. MCT4 expression is regulated by the PI3K/Akt signaling pathway and is an important regulator of cancer cell survival [97]. MCT4 expression levels are higher in HCC than in adjacent nontumor tissue, which positively correlate with tumor size. Patients with high expression levels of MCT4 had poor overall survival and time to recurrence [98]. Another study consistently showed that high MCT4 expression is associated with elevated α-fetoprotein levels and larger tumor size, as well as poor disease-free and overall survival [99]. Small-molecule MCT1 inhibitors have been developed, for example α-cyano-4-hydroxycinnamate (CHC) and AR-C155858 [100]. AZD3965 has already entered a phase 1 clinical trial in patients with advanced cancer (NCT01791595). Syrosingopine inhibits both MCT1 and MCT4, with greater potency against MCT4 (Figure 2). Syrosigopine elicits synthetic lethality with metformin. Administration of syrosingopine and metformin prevents liver-specific deletion of Tsc1 and Pten-induced liver tumor development (*Alb-Cre; Tsc1^lox/lox^; Pten^lox/lox^*) [101,102]. In most of the studies, MCT1 inhibitors were considered to block lactate uptake. Further study is necessary to evaluate the impact acetate uptake alone—excluding lactate uptake—has on tumor development.

## 7. Conclusions

Lipid metabolism is effected not only in NAFLD, but rather in a broad range of chronic liver diseases and in tumor development. A number of enzymes and metabolites are involved in reprogramming strategies of many cancer types, including hepatocellular carcinoma. Several studies with small compound inhibitors targeting catalyzing steps in selected metabolic signaling have shown convincing effects in terms of inhibiting liver cancer development and progression. To generate necessary metabolic intermediates and cellular components for rapid proliferation, cancers can use alternative pathways, such as glutamine and acetate signaling, for subsequent lipid synthesis. Therefore, it is important to systematically understand metabolic reprogramming. Identification and better understanding of cancer-specific (and disease-specific) reprogramming strategies would be important for developing stratified, more effective, and safer therapies in the future.

## Figures and Tables

**Table 1:** Overview of several clinical trials targeting lipid-metabolism-associated factors in liver diseases.

Intervention/Treatment	Condition or Disease	NCT Number	Stage of Clinical Trial	Recruitment Status	Last Update
GS-0976	NASH	NCT02856555	Phase 2	completed	11 July 2018
Selonsertib GS-0976 (firsocostat) Cilofexor	NASH	NCT03449446	Phase 2	completed	24 December 2019
Selonsertib GS-0976 (firsocostat) Cilofexor	NASH, NAFLD	NCT02781584	Phase 2	recruiting	30 June 2020
Catechin	Cirrhosis	NCT03278925	Phase 1	recruiting	29 May 2020
TVB-2640	NASH	NCT03938246	Phase 2	recruiting	09 June 2020
PF-06427878	Healthy subjects	NCT02391623	Phase 1	completed	02 March 2016
PF-06427878	Healthy subjects	NCT02855177	Phase 1	completed	04 May 2017
Aramchol	NASH	NCT02279524	Phase 2, 3	completed	26 June 2018
Aramchol	NASH	NCT04104321	Phase 3, 4	recruiting	04 November 2019
MK-8245	Type 2 Diabetes	NCT00972322	Phase 1	completed	10 September 2018
Atorvastatin	HCC	NCT03024684	Phase 4	recruiting	09 June 2020
Atorvastatin	HCC	NCT03275376	Phase 2	recruiting	11 March 2020
Pravastatin	HCC	NCT03219372	Phase 2	recruiting	29 May 2020
Sorafenib with or without pravastatin	HCC	NCT01075555	Phase 3	completed	30 March 2020
Simvastatin	Cirrhosis	NCT02968810	Phase 2	recruiting	14 May 2020

## Abbreviations

AAV	Adeno-associated viral
ACC	Acetyl-CoA carboxylase
ACLY	ATP-citrate lyase
ACP	Acyl-carrier protein
ACS	Acyl-CoA synthetase
ACSS	Short-chain acyl-CoA synthetase
ACSVL	Very long chain acyl-CoA synthetase
α-KG	α-ketoglutarate
AMPK	AMP-activated protein kinase
AST	Aspartate aminotransaminase
CDAA	Choline-deficient aminoacid-defined
CHC	α-cyano-4-hydroxycinnamate
CiC	Citrate carrier
CSC	Cancer stem cell
DEN	Diethylnitrosoamine
DGAT	Deacylglycerol acyltransferase
EGCG	Epigallocatechin gallate
ER	Endoplasmic reticulum
FA	Fatty acid
FASN	Fatty acid synthase
FPC	Fructose-palmitate-cholesterol
GLS	Glutaminase
GLUD	Glutamine dehydrogenase
GOT	Glutamic oxaloacetic transaminase
HBV	Hepatitis B virus
HCC	Hepatocellular carcinoma
HCV	Hepatitis C virus
HFD	High-fat diet
HMG	3-hydroxy-3-methylglutaryl
HMGCR	HMG-CoA reductase
HSC	Hepatic stellate cells
IDH	Isocitrate dehydrogenase
INSIG	Insulin-induced gene
LAP	Liver-enriched transcriptional activator protein
MAT	Malonyl/acetyltransferase
MCD	methionine and choline-deficient
MCT	Monocarboxylate transporter
ME	Malate enzyme
mTORC	Mammalian target of rapamycin complex
NAFLD	Nonalcoholic fatty liver disease
NASH	Nonalcoholic steatohepatitis
PPAR	Peroxisome proliferator-activated protein
SCAP	SREBP cleavage activating protein
SCD	Δ^9^-stearoyl-CoA desaturase
SLC	Solute carrier
SREBP	Sterol-regulatory element-binding protein
TCA	Tricarboxylic acid cycle
tTA	Tetracycline transactivator
